# Predictors of favorable outcome after pyloroplasty for gastroparesis: should response to pyloric dilation or Botox injection be used as a marker of surgical outcome?

**DOI:** 10.1007/s00464-023-09882-2

**Published:** 2023-02-07

**Authors:** Sven E. Eriksson, Ping Zheng, Scott Morton, Nicole Maurer, Toshitaka Hoppo, Blair A. Jobe, Shahin Ayazi

**Affiliations:** 1 Esophageal Institute, Allegheny Health Network, 4815 Liberty Avenue, Suite 439, Pittsburgh, PA 15224 USA; 2 Department of Surgery, Drexel University, Philadephia, PA USA

**Keywords:** Pyloroplasty, Gastric peroral endoscopic myotomy (G-POEM), Gastroparesis, Botox, Endoscopic dilation

## Abstract

**Introduction:**

Pyloroplasty and gastric peroral endoscopic myotomy (G-POEM) are effective surgeries for gastroparesis. The primary aim of this study was to evaluate outcomes of pyloroplasty and G-POEM in patients with gastroparesis and determine factors associated with favorable outcome. The secondary aim was to assess the utility of clinical response to preoperative pyloric dilation or botulinum toxin injection (Botox) on surgical outcome, a factor conventionally used as a favorable marker.

**Methods:**

There were 204 patients who underwent pyloroplasty (*n* = 177) or G-POEM (*n* = 27) for gastroparesis at our institution from 2014 to 2021. Demographic and clinical parameters were analyzed to assess their impact on surgical outcome. A subgroup of patients who had pyloric dilation or Botox injection were assessed separately. Favorable outcome was defined as patient reported complete resolution of the predominant gastroparesis symptom.

**Results:**

Favorable outcome was achieved in 78.4% of patients (pyloroplasty: 79.7% and G-POEM: 70.4%, *p* = 0.274). Among 61 patients where pre- and postoperative gastric emptying studies (GES) were available, mean 4-hour retention significantly improved from 33.5 to 15.0% (*p* < 0.001) and 77.0% of patients achieved normalization.

Favorable outcome was not significantly impacted by etiology of gastroparesis (*p* = 0.120), GERD (*p* = 0.518), or primary gastroparesis symptom (*p* = 0.244). Age ≥ 40 was a significant predictor of favorable surgical outcome on multivariate analysis [OR: 2.476 (1.224–5.008), *p* = 0.012]. Among the patients who had preoperative dilation (*n* = 82) or Botox injection (*n* = 46), response to these interventions was not a predictor of favorable surgical outcome (*p* = 0.192 and 0.979, respectively). However, preoperative Botox injection, regardless of response to injection, was associated with favorable surgical outcome [OR: 3.205 (CI 1.105–9.299), *p* = 0.032].

**Conclusion:**

Symptomatic improvement after pyloroplasty or G-POEM is independent of etiology of gastroparesis, GERD, and primary symptom. Response to dilation or Botox are not markers of response to surgery. However, patients who receive Botox are 3.2 times more likely to improve postoperatively.

**Graphical abstract:**

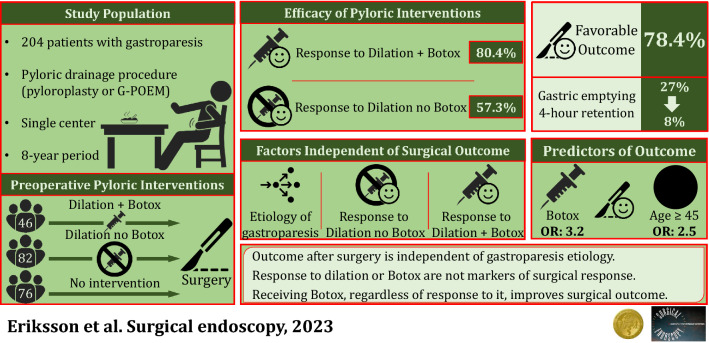

Pyloroplasty has been performed by surgeons for over 130 years, since Hieneke and Mikulicz-Radecki first described the procedure for the management of gastric outlet obstruction [1]. The adoption of pyloroplasty for the management of gastroparesis, however, was only popularized in the 21st century [2, 3]. The literature over last ten years has established pyloroplasty and gastric peroral endoscopic myotomy (G-POEM) as safe and effective surgeries for gastroparesis [4]. However, studies addressing predictive factors for a favorable surgical outcome have been limited in number, with small sample sizes and contradictory or indeterminate findings [4, 5].

Gastroparesis is characterized by delayed gastric emptying with symptoms such as nausea, vomiting, bloating, and abdominal pain in the absence of mechanical obstruction [3]. In theory, surgical management of gastroparesis is based on the underlying mechanism of dysfunction. Patients with periods of high pyloric outflow resistance, known as pylorospasm, should ideally be managed with pyloric drainage procedures, such as pyloroplasty or G-POEM [6–10]. By contrast, those with antral hypomotility as the chief mechanism of their gastroparesis would benefit the most from gastric stimulator [6–9]. In practice, however, the ability to distinguish between these phenotypes is limited by technology and a paucity of data on surgical outcome predictors.

Pyloric drainage procedures work by disrupting the pyloric sphincter, thereby reducing pyloric outflow resistance and facilitating enhanced gastric emptying [8, 11]. Pyloric botulinum toxin (Botox) injection and pneumatic dilation are thought to work by a similar mechanism [12–14]. The efficacy of dilation and Botox as stand-alone treatments is controversial [3, 15, 16]. In fact, their use is not recommended in the most recent guidelines by the American College of Gastroentology [3]. However, response to dilation and Botox are frequently used to predict surgical outcomes during patient selection [16]. This conventional practice is based on the assumption that patients who report some improvement after dilation or Botox are ‘pyloric-intervention responsive,’ and will therefore respond to pyloroplasty or G-POEM [17–22]. However, there is no evidence supporting this convention.

There is paucity of data on preoperative factors that can be reliably used to predict a favorable outcome after pyloric drainage surgery, and conventional factors such as response to dilation or Botox remain untested. Therefore, the aim of this study was to evaluate outcomes of pyloroplasty and G-POEM in patients with gastroparesis and to assess preoperative factors, such as response to pyloric dilation or botulinum toxin injection, for ability to predict surgical outcome.

## Material and methods

### Study population

This is a retrospective review of prospectively collected data from patients who underwent either laparoscopic pyloroplasty or G-POEM for the management of medically refractory gastroparesis at our institution between 2013 and 2021. Patients with at least 6-week postoperative follow-up were included for analysis. Baseline demographics, clinical information, gastroparesis etiology type (idiopathic, diabetic, or post-surgical), preoperative gastric emptying study results, and pre- and postoperative gastroparesis symptom data were collected for all patients. This study was evaluated and approved by the Institutional Review Board of Allegheny Health Network (IRB 2020-076).

### Symptom assessment

The preoperative and postoperative clinical documentation were reviewed for typical gastroparesis symptoms including postprandial fullness, early satiety, nausea/vomiting, bloating, heartburn, regurgitations, dysphagia, abdominal pain, diarrhea, and/or constipation. Preliminary analysis found that nausea/vomiting, bloating, and abdominal pain were the three most prevalent predominant symptoms, and so they were selected for analysis as potential predictors of surgical outcome.

### Surgical outcome

Patients were assessed for surgical outcome at their most recent follow-up, which was at least 6 weeks after surgery. Favorable outcome was defined as patient reported complete resolution of their predominant gastroparesis symptom. For comparative analysis, patients were divided into two groups based on whether they met criteria for favorable or unfavorable surgical outcome.

### Preoperative pyloric interventions

Among the study population, preoperative clinical courses varied based on whether patients underwent endoscopic dilation, dilation with Botox injection, or no pyloric interventions prior to surgery. This preoperative clinical course was analyzed for impact on surgical outcome. Symptomatic response to dilation or dilation with Botox was assessed at least 2 weeks following the intervention. Patients who reported symptomatic improvement were deemed ‘responders’ to their respective procedure. Patients who reported no improvement were deemed dilation or Botox non-responders, respectively. Dilation response and Botox response were analyzed for impact on surgical outcome.

### Gastric emptying scintigraphy technique and interpretation

Patients ingested a standardized meal containing 1 mCi of technetium-99m sulfur colloid. A series of anterior and posterior images were taken over the abdomen for 60 s immediately following ingestion, and then at hourly intervals for 4 h. The region containing the stomach was identified, and radiometric counts from this region immediately after ingestion were compared to the attenuation corrected counts at the hourly intervals to determine percent meal retention. Preoperative gastric emptying data were analyzed for impact on final surgical outcome in two ways: delayed vs. normal and delayed vs. severely delayed. A percent retention at 4 h ≤ 10% was considered normal, > 10% was considered delayed gastric emptying, and > 35% at was considered severely delayed gastric emptying. In a subset of patients where only the report of the ‘time-to-50%-emptying’ (T_1/2_) was available, a T_1/2_ < 90 min was considered normal and T_1/2_ ≥ 90 min was delayed. For the severity analysis in this group, a T_1/2_ ≥ 4 h was equivalent to percent retention at 4 h ≥ 50% and was considered severely delayed gastric emptying. A T_1/2_ between 90 min and 4 h was not comparable to other GES data and was excluded from severity analysis. A subset of patients who completed both preoperative and postoperative gastric emptying scintigraphy were assessed for impact of surgical intervention on GES results.

### Laparoscopic pyloroplasty technique

After placement of the ports, the pylorus was identified and mobilized to allow a tension-free closure. A 4-cm full-thickness pyloric myotomy was made extending from the antrum to the duodenum using a harmonic scalpel. A Heineke-Mikulicz pyloroplasty was then performed as previously described [2]. Endoscopy was then repeated to evaluate luminal patency and perform a leak test.

### Gastric peroral endoscopic myotomy (G-POEM) technique

The technique was identical in all patients, and all procedures were performed under general anesthesia. A submucosal cushion was created by injecting ORISE™ gel (Boston Scientific, Marlborough, MA) approximately 4 cm proximal to the pylorus along the lesser curve. A transverse mucosal incision was made with a triangle tip knife. The endoscope was advanced into the submucosal tunnel and the dissection was carried down to the pylorus. The pyloric muscular fibers were then divided. After completion, the surgical site was examined for serosal injury, irrigated, and the mucosal incision was closed with several Resolution™ clips (Boston Scientific, Marlborough, MA). Upper GI contrast study was performed on postoperative day one to assess for contrast extravasation or obstruction.

### Statistical analysis

Values for continuous variables are expressed as either mean (SD) or median with interquartile range where appropriate. Values for categorical variables are presented as frequency and percentage. Statistical analysis was performed by means of nonparametric tests, including Mann–Whitney test for difference, Pearson’s chi-square test, and Fisher’s exact test when appropriate.

Univariate logistic regression analysis of the preoperative clinical and objective data was performed to identify differences between outcome groups. Sub-analysis was performed to identify differences in outcome between idiopathic, diabetic, and post-surgical gastroparesis etiology types. A *p* value < 0.05 was considered to be statistically significant. Significant and borderline significant potential predictors in the univariate analyses were included in a multivariable logistic model stepwise selection procedure, where a predictor with significant entry level of 0.3 and significant stay level of 0.1 were selected into the model. Predictors meeting the criteria were included for multivariate analysis. All statistical analyses were performed using SAS software (version 9.4, SAS Institute, Cary, NC).

## Results

### Study population and outcomes

A total of 204 patients met inclusion criteria during the study period, of which 177 underwent pyloroplasty and 27 underwent G-POEM. Baseline demographic and clinical data for the entire population and for each procedure are shown in Table 1. At a mean (SD) follow-up of 1.27 (1.28) years, favorable outcome was achieved in 78.4% of patients. This rate was similar between pyloroplasty and G-POEM (79.7% vs. 70.4%, *p* = 0.274). Table 2 compares the outcome between two procedures. There were no major complications in the G-POEM group. Three patients were found to have suture line leaks on post-pyloroplasty upper GI series, which required reoperation and graham patch repair.

**Table 1:** Baseline demographic and clinical characteristics

Characteristic	Study population	Pyloroplasty	G-POEM	*p* Value
Age, median (IQR), years	51.0 (36.0–59.0)	51.0 (36.0–61.0)	50.0 (40.0–59.5)	0.422
Gender, n (%)				
Male	30 (14.7)	22 (12.4)	3 (12.5)	1.000
Female	174 (85.3)	155 (87.6)	24 (87.5)	
BMI, median (IQR), kg/m^2^	27.5 (23.0–33.0)	27.8 (23.0–33.0)	26.0 (23.5–33.5)	0.427
Obesity (BMI ≥ 30), n (%)	75 (36.8)	65 (36.7)	10 (37.0)	1.000
Gastroparesis type, n (%)				
Idiopathic	134 (65.7)	116 (65.5)	18 (66.7)	0.185
Diabetic	43 (21.1)	40 (22.6)	3 (11.1)	
Post-surgical	27 (13.2)	21 (11.9)	6 (22.2)	
Diabetes mellitus, n (%)	51 (25.0)	48 (27.1)	3 (11.1)	0.095
Concomitant GERD, n (%)	59 (28.9)	37 (20.9)	7 (25.9)	0.616
Pre-op delayed GES, n (%)	164 (80.5)	144 (81.4)	20 (74.1)	0.434
Severely delayed GES, n (%)	63 (34.2)	55 (53.9)	8 (29.6)	0.665
Primary symptom				
Vomiting	97 (47.6)	83 (46.9)	14 (51.9)	0.932
Bloating	22 (10.8)	19 (10.7)	3 (11.1)	
Abdominal pain	21 (10.3)	18 (10.2)	3 (11.1)	
Other	64 (31.4)	57 (32.2)	7 (25.9)	
Pre-op clinical course, n (%)				
No pre-op intervention	76 (37.3)	65 (36.7)	11 (40.7)	0.058
Botox injection	46 (22.6)	36 (20.3)	10 (37.0)	
Pneumatic dilation	82 (30.2)	76 (42.9)	6 (22.2)

**Table 2:** Surgical outcomes

Outcome	Study population	Pyloroplasty	G-POEM	*p* Value
Favorable outcome, *n* (%)	160 (78.4)	141 (79.7)	19 (70.4)	0.274
Follow-up time, mean (SD), years	1.27 (1.28)	1.32 (1.32)	1.08 (0.89)	0.288
GES normalization, *n* (%)	54 (62.8)	49 (65.3)	7 (58.3)	0.748

### Predictors of surgical outcome

The results of the univariate analysis of baseline demographic and clinical parameters with potential association with favorable surgical outcome are shown in Table 3. Favorable outcome was not significantly impacted by concomitant GERD (*p* = 0.518) or primary gastroparesis symptom (nausea/vomiting vs. bloating vs. abdominal pain, *p* = 0.244).

**Table 3:** Univariate analysis of factors predicting surgical outcome

Factor	% Favorable outcome	% Unfavorable outcome	OR (95% CI)	*p* Value
Age, median (IQR), years	50.0 (38.0–59.0)	41.0 (34.0–60.0)	1.011 (0.990–1.034)	0.305
Age, years				
≥ 40	83.1	16.9	2.341 (1.176–4.668)	0.016
< 40	67.7	32.3		
Gender				
Male	86.7	13.3	0.515 (0.170–1.564)	0.242
Female	77.0	23.0		
BMI, median (IQR), kg/m^2^	27.9 (23.2–33.0)	25.9 (20.8–32.1)	1.026 (0.978–1.076)	0.293
BMI				
≥ 30	80.0	20.0	1.160 (0.576–2.338)	0.678
< 30	77.5	22.5		
Gastroparesis etiology				
Idiopathic	74.6	25.4	–	–
Diabetic	83.7	16.3	1.749 (0.712–4.293)	0.223
Post-surgical	88.9	11.1	2.720 (0.770–9.605	0.120
Diabetes mellitus				
Yes	84.3	15.7	1.654 (0.713–3.839)	0.242
No	76.5	23.5		
Concomitant GERD				
Yes	81.4	18.6	1.286 (0.600–2.753)	0.518
No	77.2	22.8		
Preoperative GES				
Delayed	77.9	22.1	1.444 (0.519–4.023)	0.482
Normal	83.3	16.7		
Severely delayed				
Yes	84.1	15.9	1.671 (0.755–3.697)	0.205
No	15.9	84.1		
Primary symptom				
Vomiting	76.3	23.7	–	–
Bloating	90.9	9.09	3.107 (0.675–14.304)	0.146
Abdominal pain	76.2	23.8	0.995 (0.328–3.011)	0.992
Other	78.1	21.9	1.110 (0.522–2.362)	0.786

**Fig. 1:**
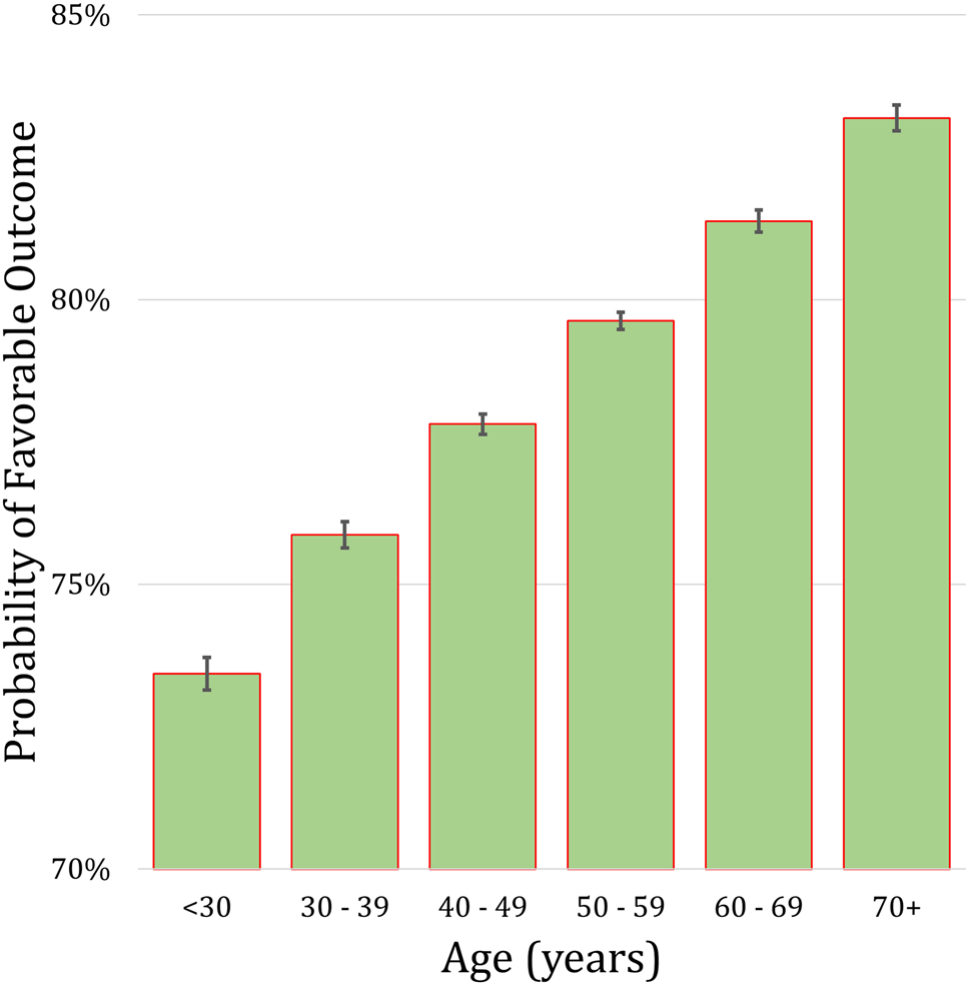
Estimated probability of favorable outcome stratified by each decade of life based on the multivariable logistic model

Patient older than 40 years were more likely to have a favorable outcome (83.1% vs. 67.7%, *p* = 0.016). The predicted probability of a favorable outcome stratified by decades of life is shown in Fig. 1.

### Preoperative pyloric interventions and outcome

A total of 82 patients underwent pyloric dilation, 46 patients underwent dilation with botulinum toxin injection, and the remaining 76 patients did not undergo any preoperative pyloric intervention. Analyses of the type of preoperative procedures and their impact on the final surgical outcome are shown in Table 4.

**Table 4:** Preoperative interventions

	% Favorable outcome	% Unfavorable outcome	OR (95% CI)	*p* Value
Pre-op clinical course, *n* (%)				
No pre-op intervention	59 (77.6)	17 (22.4)	–	–
Botox injection	41 (89.1)	5 (10.9)	2.363 (0.807–6.914)	0.117
Pneumatic dilation	60 (73.2)	22 (26.8)	0.786 (0.380–1.627)	0.516
Pre-op intervention, *n* (%)				
Botox injection	41 (89.1)	5 (10.9)	3.007 (1.053–8.584)	0.040
Pneumatic dilation	60 (73.2)	22 (26.8)		
Botox response, *n* (%)				
Botox responder	33 (89.2)	4 (10.8)	1.031 (0.101–10.530)	0.979
Botox non-responder	8 (88.9)	1 (11.1)		
Dilation response, *n* (%)				
Dilation responder	37 (78.7)	10 (21.3)	1.930 (0.719–5.182)	0.192
Dilation non-responder	23 (65.7)	12 (34.3)		

Symptomatic improvement after dilation was reported by 57.3% of patients, but this response had no impact on the final surgical outcome (*p* = 0.192). Patients who underwent dilation, regardless of symptomatic response, were not more likely to achieve favorable outcome than those who had no preoperative pyloric intervention. (*p* = 0.516). Symptomatic improvement after pneumatic dilation with Botox injection was reported by 80.4% of patients, but this response had no impact on the final surgical outcome (*p* = 0.979). Receiving Botox, regardless of symptomatic response to it, was a significant independent predictor of favorable surgical outcome on multivariate analysis (Table 5).

**Table 5:** Independent predictors of favorable outcome using multivariable logistic model

	Estimate (SE)	Odds ratio (95% CI)	*p* Value
Age ≥ 40 years	0.907 (0.360)	2.476 (1.224–5.008)	0.012
Preoperative Botox	1.165 (0.543)	3.205 (1.105–9.299)	0.032

Patients who received Botox injection at the time of pyloric dilation were more likely to experience symptomatic improvement compared to those with only dilation [OR: 3.061 (95% CI: 1.309–7.161), *p* = 0.005]. They were also more likely to achieve a favorable outcome after surgery [OR: 3.007 (95% CI: 1.053–8.568), *p* = 0.040].

### Impact of gastroparesis etiology on outcomes

**Fig. 2:**
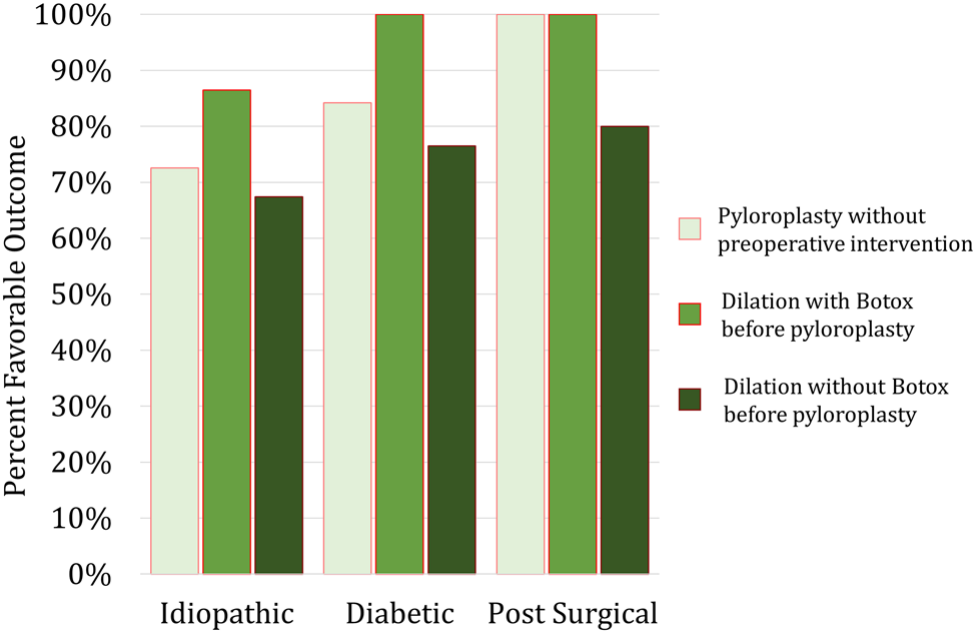
Percent favorable outcome from stratified by etiology of gastroparesis for patients who underwent pyloroplasty only, Botox with dilation before pyloroplasty and dilation without Botox before pyloroplasty. The Botox group had the highest favorable outcome rate for each etiology. However, this difference did not reach significance in the idiopathic (p = 0.126), diabetic (p = 0.411) or post-surgical (p = 0.385) gastroparesis group

There were 134 (65.7%) patients with idiopathic, 43 (21.1%) with diabetic, and 27 (13.2%) with post-surgical gastroparesis. Favorable outcome was not significantly impacted by etiology of gastroparesis (*p* = 0.120). Response to dilation (*p* = 0.284) and response to dilation with Botox (*p* = 0.121) were also similar between etiology groups. Gastroparesis etiology had no impact on favorable outcome within preoperative pyloric intervention groups (dilation with Botox, p = 0.782; dilation without Botox, *p* = 0.648; no preoperative pyloric intervention, *p* = 0.333). Figure 2 shows the rate of favorable outcome for each preoperative intervention group, stratified by etiology. Additionally, favorable outcome was not affected by response to dilation (idiopathic, *p* = 0.958; diabetic, *p* = 0.118; post-surgical, *p* = 0.316) or response to Botox (idiopathic, *p* = 0.725; diabetic, *p* = 1.000; post-surgical, *p* = 1.000) within etiology groups.

### Gastric emptying scintigraphy outcomes

**Fig. 3:**
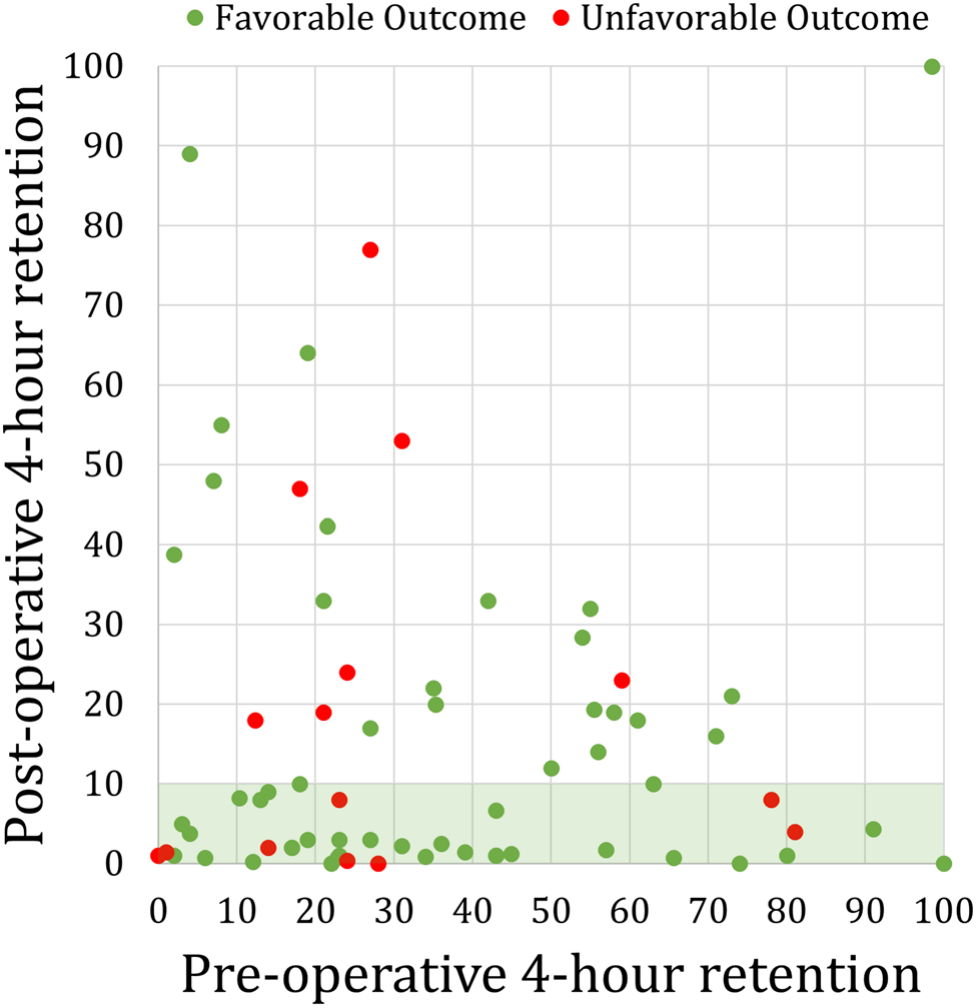
Postoperative gastric emptying result vs. preoperative gastric emptying results with favorable outcome (green) and unfavorable outcome (red) patients. Postoperative normalization (green shading) was achieved by 62.8% of patients (Color figure online)

A subset of 86 patients underwent postoperative GES. In this group, normalization of GES was achieved by 62.8% of patients. Among the 37.2% of patients who failed to normalize, 78.1% still achieved favorable clinical outcome. All factors from the analysis of surgical outcome were used in a univariate analysis of potential predictors of GES normalization, but nothing was significant. There were 61 patients who had comparable pre- and postoperative 4 h retention measurements **(**Fig. 3**)**. In this group, the median (IQR) percent retention at 4 h significantly improved from 27.0% (17.0–54.5) to 8.0% (1.3–20.5) (*p* < 0.001).

## Discussion

Gastroparesis is a challenging entity to both diagnose and treat; surgical management carries the additional challenge of appropriate candidate selection. In this study, we report the outcomes after pyloroplasty and G-POEM in a series of patients with gastroparesis at a single institution. We demonstrated that these pyloric drainage procedures result in similarly excellent outcomes. Patients improved in regard to both symptom control and gastric emptying, consistent with results reported by other centers [2, 4, 5, 11, 16, 18–20, 23, 24]. Furthermore, age older than 40 years and the use of pyloric botulinum toxin injection were found to be the factors predicting a favorable surgical outcome on multivariate analysis.

Patients who reported symptomatic improvement after preoperative pneumatic dilation with Botox, compared to those who reported no improvement, were not more likely to achieve favorable surgical outcome. The lack of impact of symptomatic response to Botox on surgical outcome runs contrary to a decade of surgical dogma. The early case series on Botox for gastroparesis demonstrated that a pyloric intervention could improve gastroparesis symptoms [25–27]. This concept influenced the early papers on the development of the G-POEM and the adaptation of pyloroplasty for gastroparesis [2, 28, 29]. However, subsequent randomized control trials failed to demonstrate the benefit of Botox, and the American College of Gastroenterology recommended against its use [3, 12, 30] Despite this recommendation, the use of Botox as a marker of surgical outcome persisted [11, 17–22]. The reasoning behind its continued use was the belief that a patient who responds to a procedure targeting the pyloric spasm would be more likely to respond to pyloric drainage surgery. Our findings demonstrate that this assumption is false. One study, frequently cited in support of this assumption, looked at 8 patients who underwent Botox prior to G-POEM and found a non-significant trend between Botox responders and favorable surgical outcome [16]. No sufficiently powered study has demonstrated this relationship with statistical significance.

The present study found that patients who were treated with pneumatic dilation with Botox injection, regardless of response, were 3.2 times more likely to have a favorable surgical outcome. A meta-analysis conducted by Mohan et al. found a somewhat similar result. They analyzed a total of 707 patients from 11 studies who underwent either pyloroplasty (*n* = 375) or G-POEM (*n* = 332). Their results showed that preoperative Botox was a significant predictor of favorable G-POEM outcome, consistent with our findings [4]. We demonstrated that Botox injection does not identify good surgical candidates, but does augment the patient’s response to surgery. A pyloric myotomy reduces outflow resistance by dividing the muscle fibers. As the myotomy heals, scar tissue forms around the pylorus and contracts, slightly diminishing the effect. Previous studies have shown that Botox prevents scar contraction by inhibiting the differentiation of fibroblasts to myofibroblasts [13, 14]. In addition, Botox-related muscle atrophy may further augment the reduction in outflow resistance [13, 14]. These findings are the likely explanation for the augmentation effect of Botox on the response to surgery that we observed in this study. Furthermore, the maximal effect of Botox lasts 3.5 months, which is longer than the median time between Botox and surgery in this study [13]. Therefore, Botox was still maximally active during the healing process in this study.

Consistent with previous studies, age, when treated as a continuous variable, was not a significant predictor of outcome [4, 11, 16, 18, 22]. However, we noticed a sharp increase in the rate of surgical outcome after 40 years of age. These patients were 2.5 times more likely to achieve favorable outcome. This is the first study to establish an association between age and surgical outcome. One explanation for this result is that gastroparesis is a slowly progressive disorder and surgical outcome may be a function of disease progression [10, 31]. Diabetes, for example, has a mean age of diagnosis > 40, and diabetic gastroparesis does not develop immediately [32]. Similar to Botox, age-related changes in fibroblast migration and growth factors responsiveness impair the development of robust postoperative scar tissue [33]. Most patients with gastroparesis are female and estrogen levels begin to decrease after age 40 [4, 34]. Estrogen modulates the inflammatory response, which results in increased wound collagen and fibronectin levels [33]. As levels naturally decline, the body’s ability to form robust scar tissue around a pyloric myotomy is further diminished.

The literature on the efficacy of pyloric dilation for the management of mechanical obstruction is plentiful, but this is not true in the setting of gastroparesis. Gourcerol et al. published the only prospective trial of dilation for gastroparesis, demonstrating a significant improvement in quality of life scores and GES in the 10 patients assessed 10 days after dilation [35]. Murray et al. found similar results in a review of 46 patients who underwent dilation for gastroparesis and found that the procedure significantly improved quality of life scores and GES with 57% of patients reporting improvement, the same rate as we report in this study [15]. However, in our study, dilation was inferior to Botox by a factor of 3, both in terms of response to the intervention itself and its ability to predict surgical outcome. Therefore, dilation should be used sparingly, and only in circumstances where Botox or prompt surgical intervention is unavailable or undesirable.

The etiology of gastroparesis had no impact on surgical outcome in the present study. This finding is particularly interesting as it was the discovery of pylorospasm in diabetic gastroparesis specifically that inspired the first pyloric interventions [10]. However, the data on the impact of gastroparesis etiology on surgical outcomes are inconsistent. Similar to our study, Rodriguez et al. did not find that etiology of gastroparesis significantly impacted the quality of life or GES in their cohort of 100 G-POEM patients. They did, however, find that patients with diabetic gastroparesis had the largest improvement in fullness/early satiety symptoms compared to their idiopathic and post-surgical counterparts [11]. By contrast, in their review of 29 patients undergoing G-POEM for gastroparesis, Gonzalez et al. found that diabetic gastroparesis was a predictor of failure at 6-month follow-up [23]. The meta-analysis by Mohan et al. found that idiopathic gastroparesis predicted postoperative GES improvement for G-POEM, but not for pyloroplasty; etiology had no impact on symptom improvement [4]. These inconsistencies in the literature highlight the need for multicenter randomized control trials to determine the true impact of gastroparesis etiology on surgical outcome.

Our study found no significant difference in outcomes between pyloroplasty and G-POEM. Similarly, a study comparing 30 laparoscopic pyloroplasty patients to 30 age, sex, and gastroparesis etiology matched G-POEM patients found that there was no difference in surgical outcome [24]. Furthermore, the aforementioned meta-analysis by Mohan et al. found no difference in outcomes between the two procedures [4]. G-POEM may be associated with slightly shorter operative times and hospital stay, but in terms of surgical outcome, laparoscopic pyloroplasty, and G-POEM are equivalent [4, 24].

We acknowledge the limitations of our study including its retrospective nature, the small G-POEM sample size, and the absence of a preoperative intervention protocol. If prompt surgery at the time of endoscopic evaluation was not desired, then Botox was typically offered. However, Botox is denied by many insurance plans and so many of these patients underwent dilation without Botox, which introduced uncontrolled socioeconomic and healthcare disparity factors. Large multicenter randomized trials are necessary control for these limitations.

## Conclusion

This study demonstrated that laparoscopic pyloroplasty and G-POEM are highly and equally effective treatments for gastroparesis, both in terms of symptom control and gastric emptying improvement. Patients over 40 were 2.5 times more likely to respond to surgical intervention. The conventional use of response to pyloric botulinum toxin injection as a marker for response to surgery was evaluated and neither response to dilation with Botox nor dilation without Botox predicted outcome. Furthermore, dilation alone was 3 times less likely than dilation with Botox to improve symptoms. Consequently, neither intervention should be used to screen surgical candidates and dilation should be used selectively. Patients who receive pneumatic dilation with Botox injection, regardless of their response to it, are 3.2 times more likely to improve after surgery, suggesting that Botox has a role in augmenting outcomes, rather than predicting them.
